# Factors correlating with serum birch pollen IgE status in pregnant women in Hokkaido, Japan: The Japan Environment and Children's Study (JECS)

**DOI:** 10.1016/j.waojou.2020.100128

**Published:** 2020-07-03

**Authors:** Yasuaki Saijo, Eiji Yoshioka, Yukihiro Sato, Toshinobu Miyamoto, Kazuo Sengoku, Yoshiya Ito, Sachiko Itoh, Chihiro Miyashita, Atsuko Araki, Reiko Kishi, the Japan Environment and Children's Study Group, Michihiro Kamijima, Shin Yamazaki, Yukihiro Ohya, Reiko Kishi, Nobuo Yaegashi, Koichi Hashimoto, Chisato Mori, Shuichi Ito, Zentaro Yamagata, Hidekuni Inadera, Takeo Nakayama, Hiroyasu Iso, Masayuki Shima, Youichi Kurozawa, Narufumi Suganuma, Koichi Kusuhara, Takahiko Katoh

**Affiliations:** a Department of Social Medicine, Asahikawa Medical University, Asahikawa, Japan; b Department of Obstetrics and Gynecology, Asahikawa Medical University, Asahikawa, Japan; c Faculty of Nursing, Japanese Red Cross Hokkaido College of Nursing, Kitami, Japan; d Center for Environmental and Health Sciences, Hokkaido University, Sapporo, Japan

**Keywords:** Birch pollen, Sensitization, IgE, Allergic rhinitis, Pregnant women, BMI, Body mass index, CI, Confidence interval, JECS, The Japan Environment and Children's Study, OR, Odds ratio

## Abstract

**Background:**

Birch pollen allergy affects pregnant women, and such allergy may affect the development of allergic diseases in their children. Using nationwide birth cohort data, this study aimed to investigate the prevalence of birch pollen IgE positivity and to identify correlating factors in pregnant women in Hokkaido, Japan, a high-latitude island.

**Methods:**

Participants included 6856 pregnant women. Participants responded to questionnaires regarding lifestyle factors and history of allergies. Data regarding parity, height, and pre-pregnancy weight were collected from medical records. Blood samples were obtained from participants during the first trimester of pregnancy, and serum allergen-specific IgE titers were determined.

**Results:**

The serum of 30.2% participants was positive for birch pollen IgE (≥0.35 UA/mL). Such positivity significantly correlated with a history of other allergic diseases, particularly food allergy and allergic rhinitis/hay fever. In multivariate logistic regression analysis, pre-pregnancy high body mass index (BMI ≥ 25) significantly correlated with birch pollen IgE positivity [odds ratio (OR), 1.24; 95% CI, 1.05–1.47; reference BMI, 18.5–24.9] and higher income (≥10 million yen per year; OR,0.55; 95% CI, 0.37–0.81; reference, household income < 2 million yen per year), and second quintile level physical activity (OR,0.75; 95% CI, 0.63–0.88; reference, the first quintile of physical activity) had significant protective effects.

**Conclusions:**

Birch pollen IgE positivity in pregnant women was positively associated with food allergy, allergic rhinitis, pre-pregnant high BMI, and was negatively associated with light exercise and high household income in Hokkaido.

**Trial registration:**

UMIN000030786.

## Introduction

Birch pollen, which causes springtime-allergy–related diseases in high-latitude countries, is one of the major causes of allergic rhinitis and a cause of asthma. Cross-reactivity with birch pollen allergens extends to plant food allergens, resulting in pollen-food allergy syndrome.^1^ Pollen-related allergic rhinitis is increasing in Japan, with Japanese cedar as the dominant pollen in much of Japan.^2^ However, its northernmost island, Hokkaido, is located at high latitude and thus has fewer Japanese cedar trees and many birch trees.^3^ The major cause of springtime allergic rhinitis on Hokkaido is birch pollen.^4^

The symptoms of allergic rhinitis can negatively affect quality of life, and they are associated with sleep disturbances. Workers with allergies may be less productive while at work, a circumstance known as presenteeism.^5^ In addition, about 70% of birch pollen allergic patients experience hypersensitivity reactions caused by IgE cross-reaction to food items,^1^ also affecting their quality of life.^6^ Serum levels of specific IgE are biomarkers for allergic reactions and can be used to assess allergy,^7^ including birch pollen sensitization.^8^ During pregnancy, allergic rhinitis and asthma can adversely affect both maternal quality of life, and, in the case of maternal asthma, perinatal outcomes.^9^

This study aims to investigate the prevalence of birch pollen IgE positivity and to identify factors that correlate with IgE status among pregnant women in a high-latitude region of Japan. Data were retrieved from the Japan Environment and Children's Study (JECS), an ongoing nationwide birth cohort study.^10^ Though JECS is a nation-wide cohort, birch pollen specific IgE among pregnant women were measured only in Hokkaido because it is the only region with a high prevalence of birch pollen allergy in Japan.

## Methods

### Participants

**Fig. 1:**
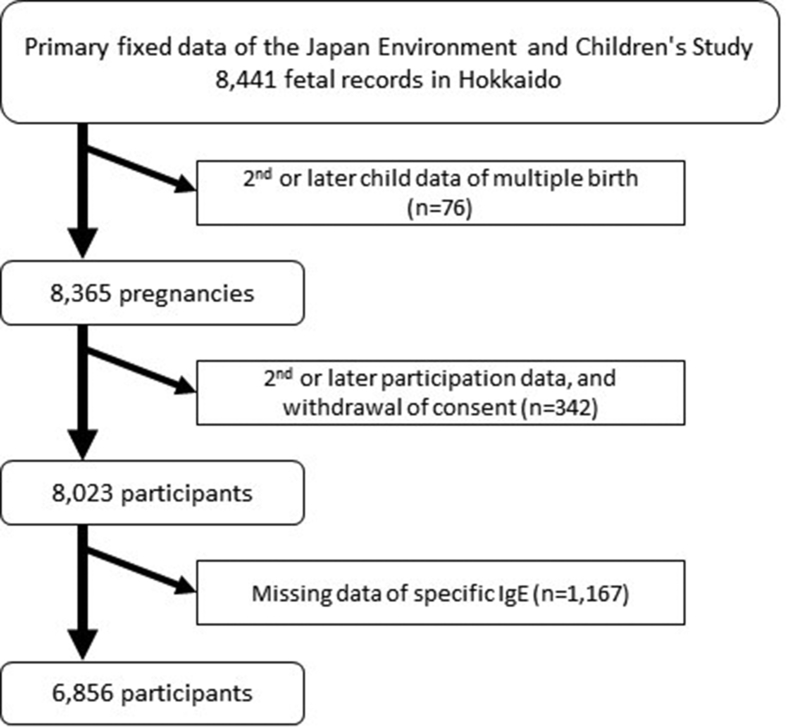
Flow diagram of study participants

The JECS is a prospective birth cohort study of participants located throughout Japan, including Hokkaido,^10^^,^^11^ and we performed a cross-sectional analysis that included pregnant women. Women were recruited in the early stages of pregnancy, and a total of 103,099 pregnancies throughout Japan were covered in this study between January 1, 2011 and March 31, 2014; discounting pregnancies in the same woman, the study involved 97,454 unique mothers. The Hokkaido unit is one of 15 regional centers of the JECS, administrating participants from 3 areas: Sapporo (Kita- and Toyohira-ward of Sapporo city; total population, 489 000), Asahikawa (Asahikawa city; population, 351 000), and Kitami area (Kitami city, Okedo town, Kunneppu town, Tsubetsu town, and Bihoro town; total population, 160,000). In Hokkaido, 8365 pregnancies (8441 fetal records) had been registered at the time of the study. After excluding second or later participation, withdrawal of consent, or missing data, we analyzed data from 6856 pregnant women (Fig. 1).

### Data collection

Questionnaires were administered to enrolled mothers during the first (T1) and second/third trimester (T2) of pregnancy. The T1 questionnaire included questions regarding age and past history of doctor-diagnosed allergy-related diseases. Participants responded to the question, “Have you ever been diagnosed by a physician for asthma, allergic rhinitis/hay fever, atopic dermatitis, allergic conjunctivitis, or food allergy (including oral allergy syndrome)?”. The mothers were also asked by research coordinators about their medication use, including steroid medications for allergy (oral, inhaled, or injected) between 12 weeks of pregnancy and submission of the T1 questionnaire. Physical activity before pregnancy was evaluated using the Japanese version (short and self-administered) of the international physical activity questionnaire (IPAQ) in the T1 questionnaire, and the physical activity in terms of Met·min/day (metabolic equivalent of a task measured as the number of minute per day) was calculated.^12^, ^13^, ^14^ Physical activity as defined in the IPAQ includes all time spent being physically active, including work-related activities, housework, and leisure-time activities. Physical activity was quintilized for categorical analysis.

The T2 questionnaire included questions related to smoking habit, drinking habit, marital status, educational attainment, household income, dog or cat residing in the home, exposure to organic solvent during pregnancy (either yes or no), and exposure to dust during pregnancy (either yes or no).

The following information was also collected from medical records: parity, gestational period, and height and pre-pregnancy weight, from which body mass index (BMI) was calculated.

### Allergen-specific IgE

Blood samples were obtained from participants during the first trimester of pregnancy if possible and during the second trimester if not. Serum allergen-specific IgE titers were determined by a contract clinical laboratory using immunological assays (ImmunoCAP, Thermo Fisher Scientific, Inc., Sweden). Specific titers were detected for the following allergens: birch, *Dermatophagoides pteronyssinus* (Der p 1), Japanese cedar, egg white, animal dander (including dog, cat, guinea pig, rat, mouse), and moth. IgE levels were classified as follows: class 1, 0.35–0.69 UA/mL; class 2, 0.70–3.49 UA/mL; class 3, 3.5–17.49 UA/mL; class 4, 17.5–49.99 UA/mL; class 5, 50–99.99 UA/mL; and class 6, ≥100 UA/mL. Positivity for allergen-specific IgE sensitization was defined as allergen-specific IgE ≥0.35 UA/mL.^7^^,^^8^^,^^15^^,^^16^

### Statistical analysis

The association between birch pollen IgE positivity and age and pregnancy term (at blood collection), pre-pregnancy BMI, smoking habits, alcohol consumption, parity, marital status, education, household income, dog and/or cat in the home, organic solvent exposure, dust exposure, steroid medication, physical activity, other allergen-specific IgEs, and past history of allergy was investigated using the chi-square test. In crude logistic regression analysis, the odds ratios (ORs) of birch pollen IgE positivity with other allergen-specific IgEs were calculated.

For participants with missing data (19.8% of the cohort), the information was replaced using multiple imputations (25 imputed datasets) based on the assumption that data were missing at random. Variables included in the imputation model were as follows: age, pregnancy term, BMI, smoking habits, alcohol consumption, parity, marital status, education, household income, dog and/or cat in the house, organic solvent exposure, dust exposure, steroid medication use, physical activity, other allergen-specific IgEs, and past history of allergy. Using the imputed datasets, the crude ORs of birch pollen IgE positivity or past history of allergy were calculated. Multiple logistic regression analysis was conducted to correlations with factors with p < 0.2 in the crude analysis of birch pollen IgE positivity.

Significance was defined as p < 0.05. All analyses were conducted using IBM SPSS Statistics 25.0 for Windows (SPSS Inc., Chicago, IL, USA) based on the dataset jecs-ag-20160424 released in June 2016 and revised in October 2016.

## Results

The distribution of each antigen-specific IgE titer is shown in Table 1. Overall, 30.2% of the cohort was positive for birch pollen IgE (≥0.35 UA/mL). Participant characteristics are compared between those who were positive and negative for birch pollen IgE in Table 2. Significant differences were found in household income and physical activity. Participant characteristics are also compared between birch pollen IgE levels (Table S1). Positivity for birch pollen IgE correlated significantly with all other antigen-specific IgEs, and Der p 1 positive had highest percentage (73.1%) of birch pollen IgE positive participants (Table 3). Positivity for birch pollen IgE correlated significantly with history of allergy of all types, and allergic rhinitis/hay fever had the highest percentage (50.0%) of birch pollen IgE positive participants (Table 4, Table S2).

**Table 1:** Distribution of allergen-specific IgE titers (n = 6856).

	(UA/mL)	<0.35	0.35–0.69	0.70–3.49	3.5–17.49	17.5–49.99	50–99.99	≥100
Birch	n	4786	217	656	735	324	105	33
	%	69.8%	3.2%	9.6%	10.7%	4.7%	1.5%	0.5%
Der p 1	n	3396	311	781	1425	726	174	43
	%	49.5%	4.5%	11.4%	20.8%	10.6%	2.5%	0.6%
Japanese cedar	n	6290	149	233	130	46	5	3
	%	91.7%	2.2%	3.4%	1.9%	0.7%	0.1%	0.0%
Egg white	n	6772	56	26	2	0	0	0
	%	98.8%	0.8%	0.4%	0.0%	0.0%	0.0%	0.0%
Animal dander	n	5186	461	754	330	89	29	7
	%	75.6%	6.7%	11.0%	4.8%	1.3%	0.4%	0.1%
Moth	n	5451	571	701	131	2	0	0
	%	79.5%	8.3%	10.2%	1.9%	0.0%	0.0%	0.0%

Moth: Bombyx mori

Animal dander: including dog, cat, guinea pig, rat, mouse.

**Table 2:** Participant characteristics according to birch pollen IgE status.

	Birch Pollen IgE status				P
	Positive (n = 2070) (≥0.35 UA/mL)		Negative (n = 4786) (<0.35 UA/mL)		
	n	%	n	%	
Age (at blood collection)					0.124
<20 y	14	0.7%	29	0.6%	
21–24	198	9.6%	413	8.6%	
25–29	628	30.3%	1378	28.8%	
30–34	763	36.9%	1743	36.4%	
35–39	395	19.1%	1053	22.0%	
≥40	71	3.4%	168	3.5%	
Missing	1	0.0%	2	0.0%	
Pregnancy term (at blood collection)					0.333
First trimester	1359	65.7%	3196	66.8%	
Second trimester	664	32.1%	1478	30.9%	
Missing	47	2.3%	112	2.3%	
Body mass index (kg/m^2^)					0.053
<18.5	337	16.3%	779	16.3%	
18.5–24.9	1469	71.0%	3486	72.8%	
≥25	239	11.5%	461	9.6%	
Missing	25	1.2%	60	1.3%	
Smoking habits					0.159
Never smoked	1046	50.5%	2335	48.8%	
Ex-smokers quitting before pregnancy	553	26.7%	1253	26.2%	
Smokers during early pregnancy	381	18.4%	972	20.3%	
Missing	90	4.3%	226	4.7%	
Alcohol consumption					0.921
Never drank	508	24.5%	1190	24.9%	
Ex-drinkers quitting before pregnancy	301	14.5%	701	14.6%	
Drinkers during early pregnancy	1172	56.6%	2680	56.0%	
Missing	89	4.3%	215	4.5%	
Parity					0.061
0	857	41.4%	1911	39.9%	
1	763	36.9%	1747	36.5%	
≥2	277	13.4%	747	15.6%	
Missing	173	8.4%	381	8.0%	
Marital status					0.819
Married	1916	92.6%	4427	92.5%	
Unmarried	96	4.6%	223	4.7%	
Divorced/widowed	17	0.8%	47	1.0%	
Missing	41	2.0%	89	1.9%	
Education (years)					0.056
<10	67	3.2%	190	4.0%	
10–12	569	27.5%	1419	29.6%	
13–16	1322	63.9%	2900	60.6%	
≥17	29	1.4%	80	1.7%	
Missing	83	4.0%	197	4.1%	
Household income (million yen per year)					0.022
<2	109	5.3%	245	5.1%	
2– <4	720	34.8%	1622	33.9%	
4– <6	628	30.3%	1501	31.4%	
6– <8	292	14.1%	642	13.4%	
8– <10	114	5.5%	244	5.1%	
≥10	47	2.3%	187	3.9%	
Missing	160	7.7%	345	7.2%	
Dog and/or cat in the house					0.236
Positive	243	11.7%	610	12.7%	
Negative	1745	84.3%	3979	83.1%	
Missing	82	4.0%	197	4.1%	
Organic solvent					0.460
Positive	20	1.0%	56	1.2%	
Negative	1958	94.6%	4519	94.4%	
Missing	92	4.4%	211	4.4%	
Dust					0.499
Positive	16	0.8%	45	0.9%	
Negative	1962	94.8%	4530	94.7%	
Missing	92	4.4%	211	4.4%	
Steroid use					0.950
Positive	8	0.4%	19	0.4%	
Negative	2001	96.7%	4627	96.7%	
Missing	61	2.9%	140	2.9%	
Physical activity (Mets · min)					0.003
≤28.3	433	20.9%	936	19.6%	
28.4–94.3	343	16.6%	983	20.5%	
94.5–205.7	394	19.0%	908	19.0%	
205.8–630.0	425	20.5%	896	18.7%	
>630.0	408	19.7%	920	19.2%	
Missing	67	3.2%	143	3.0%	

Missing categories were not used in chi-square tests.

**Table 3:** Relationship between Birch Pollen IgE status and that of other allergen-specific IgEs

	Birch Pollen IgE status				P
	Positive (n = 2070)		Negative (n = 4786)		
	n	%	n	%	
Der p 1					<0.001
Positive	1513	73.1%	1947	40.7%	
Negative	557	26.9%	2839	59.3%	
Japanese cedar					<0.001
Positive	302	14.6%	264	5.5%	
Negative	1768	85.4%	4522	94.5%	
Egg white					0.001
Positive	39	1.9%	45	0.9%	
Negative	2031	98.1%	4741	99.1%	
Animal dander					<0.001
Positive	977	47.2%	693	14.5%	
Negative	1093	52.8%	4093	85.5%	
Moth					<0.001
Positive	562	27.1%	843	17.6%	
Negative	1508	72.9%	3943	82.4%

**Table 4:** Relationship between Birch Pollen IgE status and history of physician-diagnosed allergic disease. Symptoms were missing for 91 participants

	Birch Pollen IgE status				P
	Positive (n = 2041)		Negative (n = 4724)		
	n	%	n	%	
Asthma					<0.001
Positive	390	19.1%	503	10.6%	
Negative	1651	80.9%	4221	89.4%	
Allergic rhinitis/hay fever					<0.001
Positive	1020	50.0%	1108	23.5%	
Negative	1021	50.0%	3616	76.5%	
Atopic dermatitis					<0.001
Positive	495	24.3%	772	16.3%	
Negative	1546	75.7%	3952	83.7%	
Allergic conjunctivitis					<0.001
Positive	345	16.9%	477	10.1%	
Negative	1696	83.1%	4247	89.9%	
Food allergy					<0.001
Positive	356	17.4%	225	4.8%	
Negative	1685	82.6%	4499	95.2%	

The ORs of positivity for birch pollen IgE with other allergen-specific IgEs are shown in Table S3. All antigen-specific IgEs had significantly raised ORs. Positivity for animal dander IgE had the highest OR (5.28; 95% confidence interval (CI), 4.69–5.94), followed by that of Der 1 (OR, 3.96; 95% CI, 3.54–4.43). Table S4 shows the ORs for birch pollen IgE positivity with history of allergic diseases. All allergic diseases had significantly raised ORs. The highest OR was observed for food allergy (4.20; 95% CI, 3.52–5.00), followed by that of rhinitis/hay fever (OR, 3.26; 95% CI, 2.92–3.64).

Crude ORs (Table 5) were determined for birch pollen IgE positivity and the investigated variables; multivariate adjusted ORs (Table 6) were determined for explanatory variables with p < 0.2 in the crude analysis. In multivariate analysis, statistically significant ORs were observed for BMI ≥25 (OR,1.24; 95% CI, 1.05–1.47; reference BMI, 18.5–24.9), the highest household income category (OR, 0.55; 95% CI, 0.37–0.81; reference, household income < 2 million yen per year), and the second quintile of physical activity (OR, 0.75; 95% CI, 0.63–0.88; reference: first quintile of physical activity).

**Table 5:** Crude odds ratios for birch pollen IgE positivity (multiple imputations, n = 6856).

	OR	95% CI			P
Age (at blood collection)					
<20 y	1.00				
21–24	0.99	0.51	–	1.91	0.971
25–29	0.94	0.49	–	1.79	0.848
30–34	0.90	0.47	–	1.72	0.753
35–39	0.77	0.40	–	1.48	0.436
≥40	0.87	0.43	–	1.75	0.695
Pregnancy term (at blood collection)					
First trimester	1.00				
Second trimester	1.05	0.94	–	1.18	0.360
Body mass index (kg/m^2^)					
<18.5	1.03	0.89	to	1.18	0.720
18.5–24.9	1.00				
25-	1.23	1.04	–	1.45	0.015
Smoking habits					
Never smoked	1.00				
Ex-smokers quitting before pregnancy	0.99	0.87	–	1.12	0.810
Smokers during early pregnancy	0.87	0.75	–	1.00	0.045
Alcohol consumption					
Never drank	1.00				
Ex-drinkers quitting before pregnancy	1.01	0.85	–	1.19	0.946
Drinkers during early pregnancy	1.02	0.90	–	1.16	0.754
Parity					
0	1.00				
1	0.98	0.87	–	1.10	0.673
≥2	0.87	0.72	–	1.05	0.142
Marital status					
Married	1.00				
Unmarried	0.99	0.78	–	1.27	0.963
Divorced/widowed	0.85	0.49	–	1.47	0.558
Education (years)					
<10	1.00				
10–12	1.13	0.84	–	1.50	0.421
13–16	1.29	0.97	–	1.70	0.077
≥17	1.05	0.65	–	1.71	0.844
Household income (million yen per year)					
<2	1.00				
2– <4	1.04	0.83	–	1.30	0.757
4– <6	0.96	0.77	–	1.21	0.728
6– <8	1.08	0.84	–	1.38	0.557
8– <10	1.15	0.85	–	1.54	0.361
≥10	0.57	0.39	–	0.82	0.003
Dog and/or cat in the house	0.91	0.77	–	1.07	0.236
Organic solvent	0.86	0.53	–	1.40	0.548
Dust	0.88	0.52	–	1.48	0.627
Steroid	1.00	0.53	–	1.89	0.997
Physical activity (Mets · min)					
≤28.3	1.00				
28.4–94.3	0.75	0.64	–	0.89	0.001
94.5–205.7	0.94	0.79	–	1.10	0.425
205.8–630.0	1.02	0.87	–	1.20	0.807
>630.0	0.95	0.81	–	1.12	0.573

**Table 6:** Adjusted odds ratios for birch pollen IgE positivity (multiple imputation, n = 6856)

	OR	95% CI			P
Body mass index (kg/m^2^)					
<18.5	1.03	0.89	–	1.18	0.712
18.5–24.9	1.00				
≥25	1.24	1.05	–	1.47	0.012
Smoking habits					
Never smoked	1.00				
Ex-smokers quitting before pregnancy	1.00	0.88	–	1.13	0.976
Smokers during early pregnancy	0.87	0.75	–	1.01	0.076
Parity					
0	1.00				
1	0.96	0.86	–	1.09	0.552
≥2	0.88	0.73	–	1.06	0.165
Education (years)					
<10	1.00				
10–12	1.10	0.82	–	1.47	0.535
13–16	1.26	0.94	–	1.69	0.115
≥17	1.11	0.67	–	1.84	0.681
Household income (million yen per year)					
<2	1.00				
2– <4	0.97	0.76	–	1.23	0.798
4– <6	0.90	0.70	–	1.15	0.389
6– <8	0.97	0.74	–	1.26	0.793
8– <10	0.98	0.71	–	1.35	0.901
≥10	0.55	0.37	–	0.81	0.003
Physical activity (Mets · min)					
≤28.3	1.00				
28.4–94.3	0.75	0.63	–	0.88	0.001
94.5–205.7	0.93	0.79	–	1.09	0.360
205.8–630.0	1.02	0.86	–	1.20	0.836
≥630.0	0.95	0.81	–	1.13	0.578

## Discussion

In our cohort of pregnant women in Hokkaido, 30.2% were positive for birch pollen IgE. This positivity was significantly related to history of allergic diseases, especially food allergy and allergic rhinitis/hay fever. In addition, in multivariate analysis, pre-pregnancy high BMI was significantly related to birch pollen IgE positivity, and higher income and second quintile level physical activity had significant protective effects. To our knowledge, this study is the first to report the prevalence of birch pollen IgE positivity and its related factors in a relatively large population in Japan, though our participants were limited to pregnant women. In Northern and Central Europe, over the last few decades, levels of birch pollen have risen, and the prevalence of birch pollen sensitization has also increased.^1^

A recent review reports that in general populations in Europe, the prevalence of birch pollen sensitization ranges from approximately 8%–16%.^1^ A previous study using the JECS data of participants throughout Japan reports that 55.6% were positive for Japanese cedar IgE.^15^ More than one-third of all Japanese persons have seasonal allergic rhinitis caused by Japanese cedar pollen, and the number has significantly increased in the last 2 decades.^17^ Because the prevalence of allergic rhinitis to birch pollen is unknown in the general population in Hokkaido and the prevalence of birch pollen IgE positivity was relatively high in our cohort, further studies are needed to investigate the prevalence of rhinitis and its relationship to birch pollen IgE positivity.

Sensitization to multiple antigens is common, especially among people with allergic symptoms.^18^ Among our participants who were positive for birch pollen IgE, with or without symptoms, 73% were positive for der 1-specific IgE (OR, 3.96), and 46.9% were positive for animal dander (OR, 5.28).

Oral allergy syndrome, also called pollen-food allergy syndrome, is a type of food allergy. People with birch pollen hypersensitivity may experience oral symptoms after the ingestion of apricot, peach, apple, carrot, almond, plum, hazelnut, pear, celery, fennel, parsley, aniseed, coriander, soybean, caraway, or peanut.^19^ We observed that 17.4% of the birch pollen IgE positive participants had a history of food allergy. Among allergy patients in Korea, 20% of those with a positive response to birch pollen allergen on the skin prick test had oral allergy syndrome^20^; of the participants positive for birch pollen IgE, 49.6% had a history of allergic rhinitis/hay fever, and 23.6% of participants negative for birch pollen had a history of allergic rhinitis/hay fever. Thus, positivity for a specific IgE does not necessarily indicate clinically evident allergic disease. Moreover, major causes of allergic rhinitis include other pollens, house dust, and mold,^2^ which account for a higher proportion of participants with a history of allergic rhinitis/hay fever among those negative for birch pollen.

In our present study, pre-pregnancy high BMI had a significantly higher OR for birch pollen IgE positivity. A review article reported that no clear association was found between obesity and the prevalence of allergic rhinitis or allergic conjunctivitis or increased sensitization to food allergens.^21^ However, studies in Canada and Denmark observed that obesity was significantly related to atopy sensitization (skin prick test in the Canadian study, skin prick test or specific IgE in the Danish study), especially among women;^22^^,^^23^ the later study proposed the possible influence of sex hormones on the development and expression of atopy and atopic disorders.^23^

Our multivariate analysis showed a negative correlation between the highest household income category and positivity for birch pollen IgE and no correlation between educational attainment and birch pollen IgE positivity. A systematic review reported that low socioeconomic status had a significantly higher OR for asthma, but significantly lower ORs for allergies in general, atopic dermatitis, and allergic rhinoconjunctivitis.^24^ Low educational attainment correlated significantly to atopy.^23^^,^^25^ Since the higher prevalence of allergies in higher social groups is considered consistent with the hygiene hypothesis, the protective effect of higher household income may occur by chance. Further, household income did not necessarily reflect lifetime socioeconomic status for relatively young women, and women of higher socioeconomic status may tend to have lower birch pollen exposure chance due to higher quality housing that has air conditioning, a factor known to reduce indoor pollen levels.^26^ Thus, these factors may contribute to the observed protective effect of high household income.

In our present study, the second quintile of physical activity had a significantly lower OR for birch pollen IgE positivity. Performing moderate (3–6 Mets) physical activity for 150 min/week, which is equivalent to 64 to 128 Mets·min/day, is recommended globally for substantial health benefits,^27^ and the second quintile of physical activity of our present study ranged from 28.4 to 94.3 Mets·min/day. A cohort study of children in Norway reported that low levels of physical activity in preschool children were positively associated with later atopic sensitization.^16^ However, a study of German adolescents reported that low physical activity was associated with asthma and rhinitis in boys but not girls, and that atopic sensitization was not associated with physical activity.^28^ Further, a Danish study reported that physical activity was not related to atopy sensitization.^22^ Thus, the effect of physical activity on atopic sensitization may depend on age, sex, and environmental factors. The lower OR of second quintile level physical activity may indicate that lack of physical activity is bad for atopy but that higher physical activity, particularly outdoors, increases pollen exposure.

This study has several limitations. First, since the study design is cross-sectional, we were unable to infer cause–effect relationships. Second, due to the exploratory nature of the analyses, corrections for multiple comparisons were not performed. However, all variables had significances even after Bonferroni corrections (multiplied five). Third, the participants were limited to pregnant women because we used birth cohort data. Moreover, the timing of blood sampling varied between the first and the second trimester, but the birch pollen IgE positivity had no significant difference between the first and second trimester in our present study. A study reported a statistically significant increase in birch pollen IgE over time, from preconception to postpartum, but the change was relatively small.^29^ Therefore, we believe that our result can be generalized to women of the same age group. Fourth, changes in the location of residence before participation were not considered. If someone lived in a region of Japan other than Hokkaido for many years, birch exposure would have been rare. Inclusion of many such participants would underestimate the prevalence of birch pollen IgE positivity compared to that of lifelong Hokkaido residents. Fifth, only six allergen-specific IgEs were measured, and other major allergens such as Der f 1, dog and cat fur, pollens, fungi were not evaluated because of budget limitation. However, they were selected form main asthma allergens (Der p1 and animal dander), main allergic rhinitis/hay fever allergens (cedar (all participants) and birch (only in Hokkaido), a main food allergen for children (egg white, measured as a confounder of child food allergies), and a main insect allergen in Japan (moth).^30^^,^^31^ Finally, the symptoms did not necessarily present at the study time, because allergic diseases were defined as a history of each of the allergic diseases, and seasonal variations of symptoms and specific causes of food allergies were not observed.

## Conclusions

We observed that in Hokkaido, a high-latitude island with many birch trees, 30.2% of pregnant women were positive for birch pollen IgE. Such positivity correlated significantly to a history of allergic diseases, especially food allergy (OR = 4.20) and allergic rhinitis/hay fever (OR = 3.26). Birch pollen IgE positivity correlated significantly and positively with pre-pregnancy high BMI (OR = 1.24) and negatively with higher income (OR = 0.55) and second quintile level of physical activity (OR = 0.75).

## Consent for publication

All authors agreed to the publication of the work.

## Ethics approval and consent to participate

The JECS was conducted in accordance with the Ethical Guidelines for Epidemiological Research proposed by Japan's Ministry of Health and Welfare (currently the Ministry of Health, Labour and Welfare). The JECS protocol was reviewed and approved by the Ministry of the Environment's Institutional Review Board on Epidemiological Studies and by the Ethics Committees of all participating institutions. Written informed consent was obtained from all participants.

## Authors' contributions

Research staff at Hokkaido unit centers of the JECS collected the data. The final version of the dataset (jecs-ag-20160424) was fixed and released by the Programme Office of the JECS (The National Institute for Environmental Studies). YS conducted statistical analysis of the data set. All authors contributed to the analysis of the study results. YS wrote the first draft of the manuscript. All authors approved the final version of the manuscript.

## Funding

This study was funded and supported by the 10.13039/501100006120Ministry of the Environment, Japan. The findings and conclusions of this article are solely the responsibility of the authors and do not represent the official views of the above government agency.

## Declaration of Competing Interest

The authors declare that they have no competing interests related to the contents of this article.

## Appendix B

The following individuals were members of the JECS Group as of 2019: Michihiro Kamijima (principal investigator, Nagoya City University, Nagoya, Japan), Shin Yamazaki (National Institute for Environmental Studies, Tsukuba, Japan), Yukihiro Ohya (National Center for Child Health and Development, Tokyo, Japan), Reiko Kishi (Hokkaido University, Sapporo, Japan), Nobuo Yaegashi (Tohoku University, Sendai, Japan), Koichi Hashimoto (Fukushima Medical University, Fukushima, Japan), Chisato Mori (Chiba University, Chiba, Japan), Shuichi Ito (Yokohama City University, Yokohama, Japan), Zentaro Yamagata (University of Yamanashi, Chuo, Japan), Hidekuni Inadera (University of Toyama, Toyama, Japan), Takeo Nakayama (Kyoto University, Kyoto, Japan), Hiroyasu Iso (Osaka University, Suita, Japan), Masayuki Shima (Hyogo College of Medicine, Nishinomiya, Japan), Youichi Kurozawa (Tottori University, Yonago, Japan), Narufumi Suganuma (Kochi University, Nankoku, Japan), Koichi Kusuhara (University of Occupational and Environmental Health, Kitakyushu, Japan), and Takahiko Katoh (Kumamoto University, Kumamoto, Japan).
