# Supplementary material for: Factors correlating with serum birch pollen IgE status in pregnant women in Hokkaido, Japan: The Japan Environment and Children's Study (JECS)
Source: World Allergy Organ J. 2020 Jul 3;13(6):100128. doi: 10.1016/j.waojou.2020.100128 (PMC7338638; doi:10.1016/j.waojou.2020.100128)
Supplement: Multimedia component 1 [file mmc1.docx]

Table S1 Participant characteristics according to birch pollen IgE class status

|  | Birch Pollen IgE class status | | | | | | | | | | | | | | | | |
| --- | --- | --- | --- | --- | --- | --- | --- | --- | --- | --- | --- | --- | --- | --- | --- | --- | --- |
|  | Class 0 (n=4786) | |  | Class 1 (n=217) | |  | Class 2 (n=656) | |  | Class 3 (n=735) | |  | Class 4 (n=324) | |  | Class5-6 (n=138) | |
| (UA/mL) | <0.35 | |  | 0.35-0.69 | |  | 0.70-3.49 | |  | 3.5-17.49 | |  | 17.5-49.99 | |  | >50 | |
|  | n | % |  | n | % |  | n | % |  | n | % |  | n | % |  | n | % |
| Age (at blood collection) |  |  |  |  |  |  |  |  |  |  |  |  |  |  |  |  |  |
| <20y | 29 | 0.6% |  | 2 | 0.9% |  | 3 | 0.5% |  | 3 | 0.4% |  | 2 | 0.6% |  | 4 | 2.9% |
| 21-24 | 413 | 8.6% |  | 19 | 8.8% |  | 59 | 9.0% |  | 72 | 9.8% |  | 32 | 9.9% |  | 16 | 11.6% |
| 25-29 | 1378 | 28.8% |  | 62 | 28.6% |  | 214 | 32.6% |  | 212 | 28.8% |  | 95 | 29.3% |  | 45 | 32.6% |
| 30-34 | 1743 | 36.4% |  | 85 | 39.2% |  | 226 | 34.5% |  | 279 | 38.0% |  | 125 | 38.6% |  | 48 | 34.8% |
| 35-39 | 1053 | 22.0% |  | 43 | 19.8% |  | 127 | 19.4% |  | 140 | 19.0% |  | 63 | 19.4% |  | 22 | 15.9% |
| >=40 | 168 | 3.5% |  | 6 | 2.8% |  | 27 | 4.1% |  | 28 | 3.8% |  | 7 | 2.2% |  | 3 | 2.2% |
| Missing | 2 | 0.0% |  | 0 | 0.0% |  | 0 | 0.0% |  | 1 | 0.1% |  | 0 | 0.0% |  | 0 | 0.0% |
| Pregnancy term (at blood collection) |  |  |  |  |  |  |  |  |  |  |  |  |  |  |  |  |  |
| First trimester | 3196 | 66.8% |  | 123 | 56.7% |  | 452 | 68.9% |  | 487 | 66.3% |  | 207 | 63.9% |  | 90 | 65.2% |
| Second trimester | 1478 | 30.9% |  | 89 | 41.0% |  | 194 | 29.6% |  | 225 | 30.6% |  | 108 | 33.3% |  | 48 | 34.8% |
| Missing | 112 | 2.3% |  | 5 | 2.3% |  | 10 | 1.5% |  | 23 | 3.1% |  | 9 | 2.8% |  | 0 | 0.0% |
| Body mass index (kg/m^2^) |  |  |  |  |  |  |  |  |  |  |  |  |  |  |  |  |  |
| <18.5 | 779 | 16.3% |  | 33 | 15.2% |  | 115 | 17.5% |  | 115 | 15.6% |  | 49 | 15.1% |  | 25 | 18.1% |
| 18.5–24.9 | 3486 | 72.8% |  | 147 | 67.7% |  | 460 | 70.1% |  | 531 | 72.2% |  | 232 | 71.6% |  | 99 | 71.7% |
| ≥25 | 461 | 9.6% |  | 34 | 15.7% |  | 74 | 11.3% |  | 81 | 11.0% |  | 37 | 11.4% |  | 13 | 9.4% |
| Missing | 60 | 1.3% |  | 3 | 1.4% |  | 7 | 1.1% |  | 8 | 1.1% |  | 6 | 1.9% |  | 1 | 0.7% |
| Smoking habits |  |  |  |  |  |  |  |  |  |  |  |  |  |  |  |  |  |
| Never smoked | 2335 | 48.8% |  | 105 | 48.4% |  | 321 | 48.9% |  | 376 | 51.2% |  | 177 | 54.6% |  | 67 | 48.6% |
| Ex-smokers quitting before pregnancy | 1253 | 26.2% |  | 62 | 28.6% |  | 169 | 25.8% |  | 196 | 26.7% |  | 83 | 25.6% |  | 43 | 31.2% |
| Smokers during early pregnancy | 972 | 20.3% |  | 39 | 18.0% |  | 136 | 20.7% |  | 127 | 17.3% |  | 54 | 16.7% |  | 25 | 18.1% |
| Missing | 226 | 4.7% |  | 11 | 5.1% |  | 30 | 4.6% |  | 36 | 4.9% |  | 10 | 3.1% |  | 3 | 2.2% |
| Alcohol consumption |  |  |  |  |  |  |  |  |  |  |  |  |  |  |  |  |  |
| Never drank | 1190 | 24.9% |  | 50 | 23.0% |  | 156 | 23.8% |  | 181 | 24.6% |  | 87 | 26.9% |  | 34 | 24.6% |
| Ex-drinkers quitting before pregnancy | 701 | 14.6% |  | 34 | 15.7% |  | 101 | 15.4% |  | 99 | 13.5% |  | 49 | 15.1% |  | 18 | 13.0% |
| Drinkers during early pregnancy | 2680 | 56.0% |  | 122 | 56.2% |  | 371 | 56.6% |  | 416 | 56.6% |  | 180 | 55.6% |  | 83 | 60.1% |
| Missing | 215 | 4.5% |  | 11 | 5.1% |  | 28 | 4.3% |  | 39 | 5.3% |  | 8 | 2.5% |  | 3 | 2.2% |
| Parity |  |  |  |  |  |  |  |  |  |  |  |  |  |  |  |  |  |
| 0 | 1911 | 39.9% |  | 105 | 48.4% |  | 275 | 41.9% |  | 296 | 40.3% |  | 135 | 41.7% |  | 46 | 33.3% |
| 1 | 1747 | 36.5% |  | 67 | 30.9% |  | 246 | 37.5% |  | 280 | 38.1% |  | 113 | 34.9% |  | 57 | 41.3% |
| >2 | 747 | 15.6% |  | 28 | 12.9% |  | 87 | 13.3% |  | 95 | 12.9% |  | 42 | 13.0% |  | 25 | 18.1% |
| Missing | 381 | 8.0% |  | 17 | 7.8% |  | 48 | 7.3% |  | 64 | 8.7% |  | 34 | 10.5% |  | 10 | 7.2% |
| Marital status |  |  |  |  |  |  |  |  |  |  |  |  |  |  |  |  |  |
| Married | 4427 | 92.5% |  | 202 | 93.1% |  | 598 | 91.2% |  | 687 | 93.5% |  | 305 | 94.1% |  | 124 | 89.9% |
| Unmarried | 223 | 4.7% |  | 9 | 4.1% |  | 40 | 6.1% |  | 27 | 3.7% |  | 12 | 3.7% |  | 8 | 5.8% |
| Divorced/widowed | 47 | 1.0% |  | 2 | 0.9% |  | 3 | 0.5% |  | 8 | 1.1% |  | 2 | 0.6% |  | 2 | 1.4% |
| Missing | 89 | 1.9% |  | 4 | 1.8% |  | 15 | 2.3% |  | 13 | 1.8% |  | 5 | 1.5% |  | 4 | 2.9% |
| Education (years) |  |  |  |  |  |  |  |  |  |  |  |  |  |  |  |  |  |
| <10 | 190 | 4.0% |  | 5 | 2.3% |  | 21 | 3.2% |  | 26 | 3.5% |  | 12 | 3.7% |  | 3 | 2.2% |
| 10-12 | 1419 | 29.6% |  | 60 | 27.6% |  | 192 | 29.3% |  | 192 | 26.1% |  | 91 | 28.1% |  | 34 | 24.6% |
| 13-16 | 2900 | 60.6% |  | 136 | 62.7% |  | 411 | 62.7% |  | 471 | 64.1% |  | 209 | 64.5% |  | 95 | 68.8% |
| >17 | 80 | 1.7% |  | 5 | 2.3% |  | 5 | 0.8% |  | 13 | 1.8% |  | 4 | 1.2% |  | 2 | 1.4% |
| Missing | 197 | 4.1% |  | 11 | 5.1% |  | 27 | 4.1% |  | 33 | 4.5% |  | 8 | 2.5% |  | 4 | 2.9% |
| Household income (million yen per year) |  |  |  |  |  |  |  |  |  |  |  |  |  |  |  |  |  |
| <2 | 245 | 5.1% |  | 14 | 6.5% |  | 37 | 5.6% |  | 36 | 4.9% |  | 12 | 3.7% |  | 10 | 7.2% |
| 2- <4 | 1622 | 33.9% |  | 75 | 34.6% |  | 234 | 35.7% |  | 255 | 34.7% |  | 100 | 30.9% |  | 56 | 40.6% |
| 4- <6 | 1501 | 31.4% |  | 65 | 30.0% |  | 200 | 30.5% |  | 216 | 29.4% |  | 103 | 31.8% |  | 44 | 31.9% |
| 6- <8 | 642 | 13.4% |  | 27 | 12.4% |  | 84 | 12.8% |  | 116 | 15.8% |  | 52 | 16.0% |  | 13 | 9.4% |
| 8- <10 | 244 | 5.1% |  | 10 | 4.6% |  | 28 | 4.3% |  | 45 | 6.1% |  | 24 | 7.4% |  | 7 | 5.1% |
| >10 | 187 | 3.9% |  | 6 | 2.8% |  | 19 | 2.9% |  | 14 | 1.9% |  | 8 | 2.5% |  | 0 | 0.0% |
| Missing | 345 | 7.2% |  | 20 | 9.2% |  | 54 | 8.2% |  | 53 | 7.2% |  | 25 | 7.7% |  | 8 | 5.8% |
| Dog and/or cat in the house |  |  |  |  |  |  |  |  |  |  |  |  |  |  |  |  |  |
| Positive | 610 | 12.7% |  | 26 | 12.0% |  | 81 | 12.3% |  | 87 | 11.8% |  | 35 | 10.8% |  | 14 | 10.1% |
| Negative | 3979 | 83.1% |  | 180 | 82.9% |  | 548 | 83.5% |  | 614 | 83.5% |  | 281 | 86.7% |  | 122 | 88.4% |
| Missing | 197 | 4.1% |  | 11 | 5.1% |  | 27 | 4.1% |  | 34 | 4.6% |  | 8 | 2.5% |  | 2 | 1.4% |
| Organic solvent |  |  |  |  |  |  |  |  |  |  |  |  |  |  |  |  |  |
| Positive | 56 | 1.2% |  | 3 | 1.4% |  | 7 | 1.1% |  | 6 | 0.8% |  | 4 | 1.2% |  | 0 | 0.0% |
| Negative | 4519 | 94.4% |  | 203 | 93.5% |  | 619 | 94.4% |  | 693 | 94.3% |  | 310 | 95.7% |  | 133 | 96.4% |
| Missing | 211 | 4.4% |  | 11 | 5.1% |  | 30 | 4.6% |  | 36 | 4.9% |  | 10 | 3.1% |  | 5 | 3.6% |
| Dust |  |  |  |  |  |  |  |  |  |  |  |  |  |  |  |  |  |
| Positive | 45 | 0.9% |  | 3 | 1.4% |  | 3 | 0.5% |  | 6 | 0.8% |  | 3 | 0.9% |  | 1 | 0.7% |
| Negative | 4530 | 94.7% |  | 203 | 93.5% |  | 623 | 95.0% |  | 693 | 94.3% |  | 311 | 96.0% |  | 132 | 95.7% |
| Missing | 211 | 4.4% |  | 11 | 5.1% |  | 30 | 4.6% |  | 36 | 4.9% |  | 10 | 3.1% |  | 5 | 3.6% |
| Steroid use |  |  |  |  |  |  |  |  |  |  |  |  |  |  |  |  |  |
| Positive | 19 | 0.4% |  | 2 | 0.9% |  | 2 | 0.3% |  | 1 | 0.1% |  | 2 | 0.6% |  | 1 | 0.7% |
| Negative | 4627 | 96.7% |  | 211 | 97.2% |  | 635 | 96.8% |  | 709 | 96.5% |  | 311 | 96.0% |  | 135 | 97.8% |
| Missing | 140 | 2.9% |  | 4 | 1.8% |  | 19 | 2.9% |  | 25 | 3.4% |  | 11 | 3.4% |  | 2 | 1.4% |
| Physical activity (Mets · min) |  |  |  |  |  |  |  |  |  |  |  |  |  |  |  |  |  |
| <28.3 | 936 | 19.6% |  | 53 | 24.4% |  | 129 | 19.7% |  | 157 | 21.4% |  | 67 | 20.7% |  | 27 | 19.6% |
| 28.4–94.3 | 983 | 20.5% |  | 34 | 15.7% |  | 108 | 16.5% |  | 121 | 16.5% |  | 61 | 18.8% |  | 19 | 13.8% |
| 94.5–205.7 | 908 | 19.0% |  | 40 | 18.4% |  | 128 | 19.5% |  | 136 | 18.5% |  | 66 | 20.4% |  | 24 | 17.4% |
| 205.8–630.0 | 896 | 18.7% |  | 39 | 18.0% |  | 134 | 20.4% |  | 164 | 22.3% |  | 56 | 17.3% |  | 32 | 23.2% |
| >630.0 | 920 | 19.2% |  | 43 | 19.8% |  | 136 | 20.7% |  | 134 | 18.2% |  | 63 | 19.4% |  | 32 | 23.2% |
| Missing | 143 | 3.0% |  | 8 | 3.7% |  | 21 | 3.2% |  | 23 | 3.1% |  | 11 | 3.4% |  | 4 | 2.9% |

Table S2 Relationship between Birch Pollen IgE class status and history of allergic disease

|  | Birch Pollen IgE class status | | | | | | | | | | | | | | | | | | P | |
| --- | --- | --- | --- | --- | --- | --- | --- | --- | --- | --- | --- | --- | --- | --- | --- | --- | --- | --- | --- | --- |
|  | Class 0 (n=4724) | |  | Class 1 (n=213) | |  | Class 2 (n=646) | |  | Class 3 (n=726) | |  | Class 4 (n=319) | |  | Class5-6 (n=137) | |  | |  |
|  | <0.35 | |  | 0.35-0.69 | |  | 0.70-3.49 | |  | 3.5-17.49 | |  | 17.5-49.99 | |  | >50 | |  | |  |
|  | n | % |  | n | % |  | n | % |  | n | % |  | n | % |  | n | % |  | |  |
| Asthma |  |  |  |  |  |  |  |  |  |  |  |  |  |  |  |  |  | <0.001 | |  |
| Positive | 503 | 10.6% |  | 39 | 18.3% |  | 137 | 21.2% |  | 129 | 17.8% |  | 60 | 18.8% |  | 25 | 18.2% |  | |  |
| Negative | 4221 | 89.4% |  | 174 | 81.7% |  | 509 | 78.8% |  | 597 | 82.2% |  | 259 | 81.2% |  | 112 | 81.8% |  | |  |
| Allergic rhinitis/hay fever |  |  |  |  |  |  |  |  |  |  |  |  |  |  |  |  |  | <0.001 | |  |
| Positive | 1108 | 23.5% |  | 68 | 31.9% |  | 270 | 41.8% |  | 380 | 52.3% |  | 205 | 64.3% |  | 97 | 70.8% |  | |  |
| Negative | 3616 | 76.5% |  | 145 | 68.1% |  | 376 | 58.2% |  | 346 | 47.7% |  | 114 | 35.7% |  | 40 | 29.2% |  | |  |
| Atopic dermatitis |  |  |  |  |  |  |  |  |  |  |  |  |  |  |  |  |  | <0.001 | |  |
| Positive | 772 | 16.3% |  | 54 | 25.4% |  | 145 | 22.4% |  | 188 | 25.9% |  | 70 | 21.9% |  | 38 | 27.7% |  | |  |
| Negative | 3952 | 83.7% |  | 159 | 74.6% |  | 501 | 77.6% |  | 538 | 74.1% |  | 249 | 78.1% |  | 99 | 72.3% |  | |  |
| Allergic conjunctivitis |  |  |  |  |  |  |  |  |  |  |  |  |  |  |  |  |  | <0.001 | |  |
| Positive | 477 | 10.1% |  | 35 | 16.4% |  | 105 | 16.3% |  | 112 | 15.4% |  | 59 | 18.5% |  | 34 | 24.8% |  | |  |
| Negative | 4247 | 89.9% |  | 178 | 83.6% |  | 541 | 83.7% |  | 614 | 84.6% |  | 260 | 81.5% |  | 103 | 75.2% |  | |  |
| Food allergy |  |  |  |  |  |  |  |  |  |  |  |  |  |  |  |  |  | <0.001 | |  |
| Positive | 225 | 4.8% |  | 21 | 9.9% |  | 53 | 8.2% |  | 128 | 17.6% |  | 97 | 30.4% |  | 57 | 41.6% |  | |  |
| Negative | 4499 | 95.2% |  | 192 | 90.1% |  | 593 | 91.8% |  | 598 | 82.4% |  | 222 | 69.6% |  | 80 | 58.4% |  | |  |

Symptoms were missing for 91 participants.

Table S3. Odds ratios of birch pollen IgE positivity with positivity for other IgEs (n = 6856)

| IgE | OR | 95% CI | | | P |
| --- | --- | --- | --- | --- | --- |
| Der p 1 | 3.96 | 3.54 | – | 4.43 | <0.001 |
| Japanese cedar | 2.93 | 2.46 | – | 3.48 | <0.001 |
| Egg white | 2.02 | 1.31 | – | 3.12 | 0.001 |
| Animal dander | 5.28 | 4.69 | – | 5.94 | <0.001 |
| Moth | 1.74 | 1.54 | – | 1.97 | <0.001 |

Table S4 Odds ratios of positive levels of specific IgE to birch pollen for physician-diagnosed allergic diseases (multiple imputation, n = 6856)

| Outcomes | OR | 95% CI | | | P |
| --- | --- | --- | --- | --- | --- |
| Asthma | 1.97 | 1.70 | – | 2.28 | <0.001 |
| Allergic rhinitis/hay fever | 3.26 | 2.92 | – | 3.64 | <0.001 |
| Atopic dermatitis | 1.64 | 1.44 | – | 1.86 | <0.001 |
| Allergic conjunctivitis | 1.81 | 1.55 | – | 2.10 | <0.001 |
| Food allergy | 4.20 | 3.52 | – | 5.00 | <0.001 |
